# Prognostic and predictive imaging markers of hepatocellular carcinoma: a pictorial essay

**DOI:** 10.1186/s13244-025-02058-7

**Published:** 2025-08-15

**Authors:** Claudia Deyirmendjian, Banmeet Padda, Kathryn J. Fowler, Victoria Chernyak, Claude B. Sirlin, Hanyu Jiang, Kim-Nhien Vu, Joseph R. Dadour, Jessica Murphy-Lavallée, Jean-Sébastien Billiard, Damien Olivié, Bich N. Nguyen, An Tang

**Affiliations:** 1 Department of Radiology, Radiation Oncology, and Nuclear Medicine, Université de Montréal, Montréal, Québec Canada; 2 Liver Imaging Group, Department of Radiology, University of California San Diego, San Diego, CA USA; 3 Department of Radiology, Columbia University Irving Medical Center, New York, NY USA; 4 Department of Radiology, West China Hospital, Chengdu, China; 5 Centre de recherche du Centre hospitalier de l’Université de Montréal (CRCHUM), Montréal, Québec Canada; 6 Department of Radiology, Centre hospitalier de l’Université de Montréal (CHUM), Montréal, Québec Canada; 7 Service of Pathology, Centre hospitalier de l’Université de Montréal (CHUM), Montréal, Québec Canada

**Keywords:** Carcinoma (hepatocellular), Carcinogenesis, Magnetic resonance imaging, Tomography (X-ray computed)

## Abstract

**Abstract:**

Hepatocellular carcinoma (HCC) encompasses a wide array of histopathologic and genetic features that can be broadly categorized as proliferative or non-proliferative HCC to reflect tumor aggressiveness. However, accurately characterizing tumor behavior remains challenging due to the biologic heterogeneity of HCC and limited access to tissue samples. Currently, imaging is used for the diagnosis of HCC using the Liver Imaging Reporting and Data System (LI-RADS) without histologic confirmation in most cases. Emerging data suggest that imaging can provide clinical insight beyond diagnosis and predict patient outcomes by identifying key prognostic features, including those not yet integrated in LI-RADS. Certain CT and MRI features correlate with proliferative and non-proliferative HCC, and may yield prognostic information. Imaging findings such as tumor size, multifocality, and low apparent diffusion coefficient (ADC) have also been associated with microvascular invasion—an independent marker of poor prognosis. Growing data support the role of imaging in predicting treatment responsiveness before therapy initiation, which may influence the selection of a therapeutic agent. The radiologist can offer key clinical information by understanding and describing the prognostic and predictive features in HCC imaging.

**Critical relevance statement:**

This study provides radiologists with a comprehensive summary of imaging findings associated with HCC prognosis, treatment responsiveness, and microvascular invasion.

**Key Points:**

- Hepatocellular carcinoma (HCC) is a heterogeneous cancer leading to challenges in diagnosis and management.
- Tumors can exhibit imaging features associated with proliferative or non-proliferative HCC.
- Key imaging features can help predict tumor aggressiveness and treatment responsiveness before the therapy is applied.
- Further research leveraging molecular data and applying machine learning models can improve our understanding of HCC prognostication.

**Graphical Abstract:**

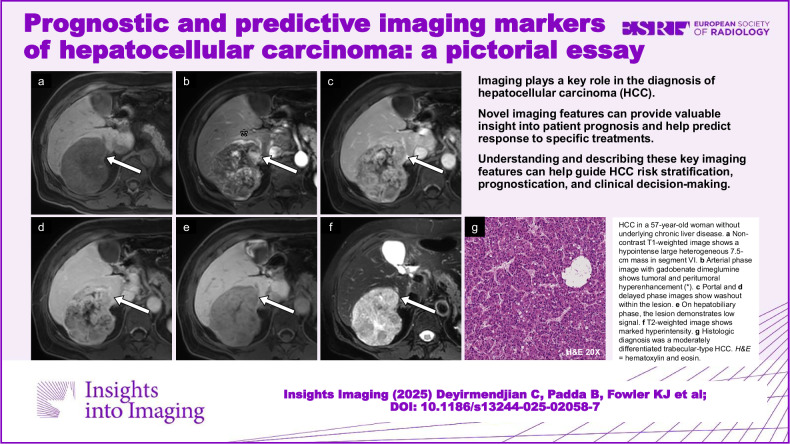

## Introduction

Liver cancer represents the third most common cause of cancer-related mortality and is the sixth most frequently diagnosed cancer worldwide. Globally, over 860,000 incident cases of liver cancer were diagnosed, with over 750,000 deaths annually in 2022 [1]. Hepatocellular carcinoma (HCC) is the most common type of primary liver cancer [2]. Although survival has improved over time, the prognosis is guarded with HCC-specific mortality exceeding 50% at 5 years [3]. The pathophysiology of HCC is multifactorial and involves genetic and microenvironmental insults to hepatic parenchyma.

Diagnostic criteria for HCC can be met on the basis of imaging findings using the Liver Imaging Reporting and Data System (LI-RADS) [4]. LI-RADS offers standardized algorithms to determine the probability of malignancy and HCC using features such as tumor size, arterial phase hyperenhancement, nonperipheral washout, enhancing capsule, and threshold growth. However, LI-RADS currently provides limited information on patient outcomes at the time of diagnosis.

Classifying HCC according to suspected tumor aggressiveness can influence treatment selection, surveillance plans, and patient expectations. Considering the significant biologic heterogeneity of HCC and the wide range of clinical outcomes, accurate stratification of HCC remains difficult. Histopathologic and molecular features are highly useful adjuncts in HCC characterization and are essential for diagnosis when radiological signs are equivocal. Unfortunately, as the role of biopsy in the workup of HCC has decreased, so has the availability of microscopic markers of prognosis. Consequently, there is a growing need to identify prognostic markers without resorting to invasive techniques.

Through correlation with histopathologic, molecular, and imaging features, emerging data suggest that imaging can provide important information on prognostic and predictive markers. Prognostic markers predict the natural evolution of disease. Predictive markers offer insight into potential responsiveness to treatment, which could influence therapy selection. The objectives of the current review are (1) to describe known prognostic and predictive markers of HCC, (2) to understand the imaging features associated with HCC prognosis, and (3) to identify these features on CT and MRI.

## Proliferative and non-proliferative classification

Hepatocarcinogenesis is influenced by an amalgam of genetic pathways that result in differences in tumor aggressiveness. Genetic alterations activate oncogenic signaling pathways and this translates in the expression of specific markers, providing insights on tumor cell proliferation and differentiation status.

**Fig. 1:**
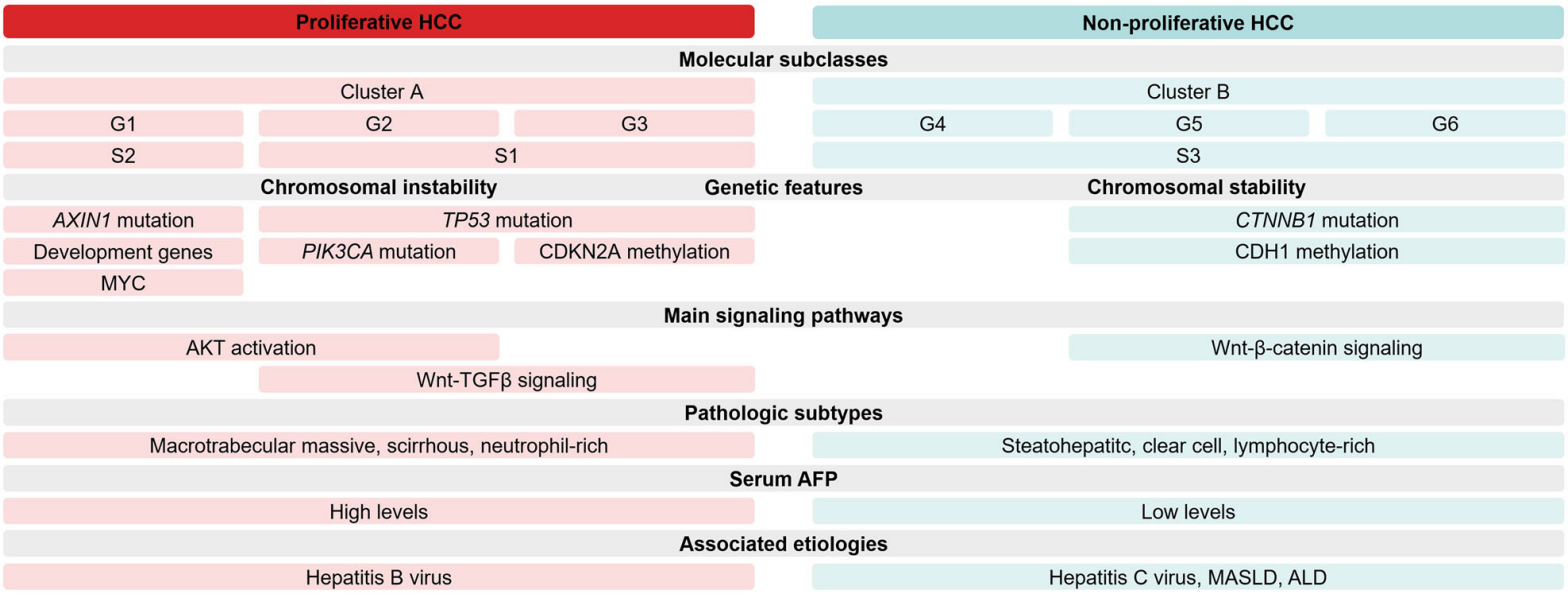
Examples of the molecular, genetic, pathologic, clinical, and serum markers in proliferative and non-proliferative HCC. AFP, alpha-fetoprotein; AKT, protein kinase B; ALD, alcohol-associated liver disease; AXIN1, axis inhibitor 1; CDH1, cadherin 1; CDKN2A, cyclin-dependent kinase inhibitor 2A; CTNNB1, catenin beta-1; MASLD, metabolic dysfunction-associated steatotic liver disease; PIK3CA, phosphoinositide-3-kinase-catalytic-alpha; TGF-β, transforming growth factor-β; TP53, tumor protein p53

While it is challenging to define neat categories of HCC given the extensive genomic alterations, classification of HCC is necessary for risk stratification, prognostication, and clinical decision-making. To better reflect the behaviors of different HCC phenotypes, HCC can be divided into two broad subgroups: proliferative HCC and non-proliferative HCC. Each subgroup is characterized by various molecular, histopathologic, clinical, and serum findings (Fig. 1) [5].

The proliferative subtype is defined by excessive genomic and epigenetic changes associated with poor prognosis and worse survival outcomes. Classification by Lee et al in 2004 defined clusters based on cell proliferation and cell survival, with proliferative HCC aligning with Cluster A, which exhibits high levels of cell proliferation [6]. Proliferative HCC can also be characterized by chromosomal instability or its predominant signaling pathways. High vascular invasion is a feature of proliferative HCC likely driven by numerous genetic and epigenetic properties, such as the MYC oncogene activation, which upregulates vascular invasion-related mRNA and microRNA [7, 8]. Cellular dedifferentiation and increased epithelial-mesenchymal transition promote angiogenesis and vascular invasion via signaling pathways including transforming growth factor-β (TGF-β) [9]. The main molecular classes of HCC are summarized in Table 1.

**Table 1:** Main molecular classification systems of HCC

Lee et al [6]	Boyault et al [10]	Hoshida et al [7]
▪ Classes: Cluster A and B ▪ Classification based on tumor proliferation level and cell survival. ▪ Cluster A tumors generally demonstrate higher cell proliferation, and Cluster B tumors demonstrate lower cell proliferation.	▪ Classes: G1-G6 ▪ Classification based on 16 genes and clinical data. ▪ Two large tumor groups are associated with chromosome instability (G1-G3) or chromosome stability (G4-G6).	▪ Classes: S1-S3 ▪ Proliferative S1 class is characterized by Wnt/TGF-β activity. ▪ Proliferative S2 class is linked to MYC activity. ▪ Non-proliferative S3 class is associated with well-differentiated tumors.

On the other hand, non-proliferative HCC is a less aggressive phenotype associated with better outcomes. Tumor cells are generally more differentiated than proliferative type HCC, and they have a tendency to retain hepatocyte-like features. Non-proliferative HCC correlates with subtypes defined by low cell proliferation and chromosomal stability. They tend to progress less rapidly through the Wnt-β-catenin signaling pathway activation and the *CTNNB1* mutation, a commonly mutated gene in HCC associated with a favorable prognosis [6, 10, 11]. Frequently, HCC tumors do not fall neatly into a single proliferative or non-proliferative category but exhibit a mixture of features.

Molecular markers have a growing role in the management of HCC, and recent studies support that understanding genetic changes may help guide therapy. For example, Ziv et al analyzed the gene mutation signature of HCC and non-HCC liver tumors treated with transarterial embolization [12]. The most predictive genes were associated with Wnt-β-catenin and hypoxia signaling pathways, such as *CTNNB1*, a downstream activator of Wnt-β-catenin signaling. A dependence on glycolysis upregulation, which has been linked to the involved pathways, was a proposed mechanism for the increased susceptibility to ischemia. Understanding the pathophysiology and classification of HCC allows radiologists to better assess and potentially treat HCC.

## Pathologic classification

Most HCCs are classified as not otherwise specified (NOS) conventional type with one of the following principal growth patterns: (1) trabecular, (2) pseudoglandular, (3) solid, and (4) macrotrabecular.

**Fig. 2:**
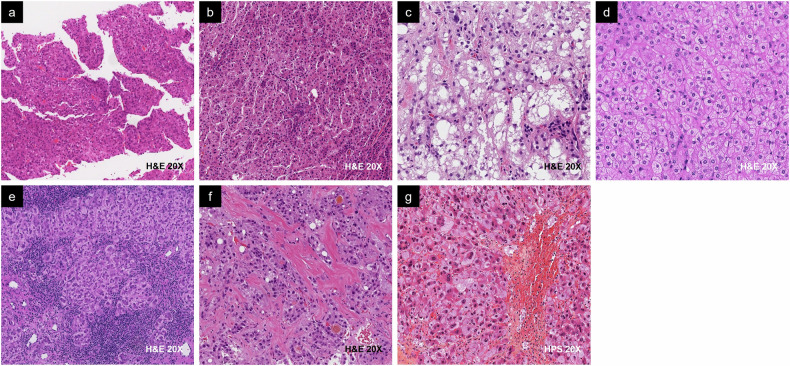
Examples of the WHO Classification of HCC subtypes. **a** Macrotrabecular massive HCC is associated with proliferative HCC and generally has a worse prognosis than (**b**) not otherwise specified (NOS) conventional HCC, in this case trabecular. **c** Steatohepatitic, (**d**) clear cell, and (**e**) lymphocyte-rich HCC are associated with non-proliferative HCC and generally have a better prognosis compared to NOS conventional HCC. **f** Fibrolamellar and (**g**) chromophobe HCC are not clearly associated with either class but generally have a similar prognosis to NOS conventional HCC. Other pathologic subtypes (not shown) include neutrophil-rich and scirrhous HCC, which are typically associated with proliferative HCC. H&E, hematoxylin and eosin; HPS, hematoxylin phloxine saffron

As molecular evidence becomes more widely available, the 2019 World Health Organization (WHO) has refined its histologic classification and named eight special HCC variants, according to their distinct prognoses: macrotrabecular massive, scirrhous, neutrophil-rich, steatohepatitic, clear cell, lymphocyte-rich, fibrolamellar, and chromophobe (Fig. 2 and Table 2) [13, 14]. Approximately 35% of HCCs meet the criteria for a special histologic subtype.

**Table 2:** WHO Classification of pathologic subtypes [14, 16]

	WHO subtype	Frequency	Description
Proliferative HCC			
	Macrotrabecular massive	< 5%	▪ Predominant macrotrabecular growth pattern (> 10 cells in thickness) in > 50% tumor ▪ Vascular invasion is common
	Scirrhous	4%	▪ More than 50% of tumor shows dense intratumoral fibrosis ▪ Peripheral tumor cells resembling hepatic stem/progenitor cells
	Neutrophil-rich	< 1%	▪ Numerous and diffuse neutrophilic tumor infiltration, may have sarcomatoid areas
Non-proliferative HCC			
	Steatohepatitic	5–20%	▪ Fat accumulation, ballooning of tumor cells, Mallory-Denk bodies, pericellular fibrosis and intratumoral inflammation
	Clear cell	3–7%	▪ Greater than 80% of tumor cells show abundant clear cell cytoplasm from glycogen accumulation
	Lymphocyte-rich	< 1%	▪ Prominent intratumoral lymphocytic infiltrate (T cells CD8+) that outnumbers tumor cells
Unclear prognosis			
	Fibrolamellar	1%	▪ Large eosinophilic tumor cells with prominent nucleoli and abundant intratumoral lamellated fibrosis ▪ Arising in non-cirrhotic liver
	Chromophobe	3%	▪ Chromophobic cytoplasm characterized by light, almost clear cytoplasm and mainly bland tumor nuclei, but focal areas of striking nuclear atypia

Among the different subtypes, some demonstrate features and behaviors that reflect proliferative or non-proliferative HCC. Proliferative HCC has been associated with macrotrabecular massive, neutrophil-rich, and scirrhous HCC, although the prognosis of the latter is still controversial in the current literature [15]. A macrotrabecular growth pattern is defined as trabeculae > 10 cells in thickness. Macrotrabecular massive HCC is a distinct entity characterized by a predominantly macrotrabecular growth pattern representing more than 50% of the entire tumor.

Conversely, non-proliferative HCC has been associated with steatohepatitic, clear cell, and lymphocyte-rich HCC [15]. Steatohepatitic HCC is the most common special subtype with an incidence of 5–20%. It is defined by fat accumulation, ballooning of tumor cells, Mallory-Denk bodies, pericellular fibrosis, and intratumoral inflammation. On MRI, about 80% are associated with intralesional fatty changes [16].

Fibrolamellar and chromophobe subtypes are rare subtypes with unclear prognosis.

In many cases, the pathologic subtype alone does not provide reliable prognostic information. While the histopathologic diagnosis contributes to our understanding of tumor behavior, it remains important to consider its prognostic value in the context of other findings, for example, tumor size, liver function, and physical status [17].

## Clinical and biochemical prognostic features

The major risk factor for HCC is liver cirrhosis. Specific clinical etiologies, including infectious and metabolic conditions, have been associated with HCC prognosis. Hepatitis B virus (HBV)-induced HCC is more common in Asian populations, while hepatitis C virus (HCV) is the leading risk factor for HCC in the Western population [18]. Due to its integration into the host DNA, HBV infection causes oncogenic alterations linked to high proliferative potential, whereas HCV infection is not associated with a strong oncogenic effect [18]. Other clinical etiologies associated with non-proliferative HCC include metabolic dysfunction-associated steatotic liver disease (MASLD), its more advanced form, metabolic dysfunction-associated steatohepatitis (MASH), and alcohol-related liver disease (ALD) [18]. Serum markers of HCC are being actively studied for their prognostic value. Alpha-fetoprotein (AFP) and des-γ-carboxy prothrombin (DCP) are two established serum markers, and higher levels have been linked to poorer prognosis [19].

## Prognostic imaging features

**Fig. 3:**
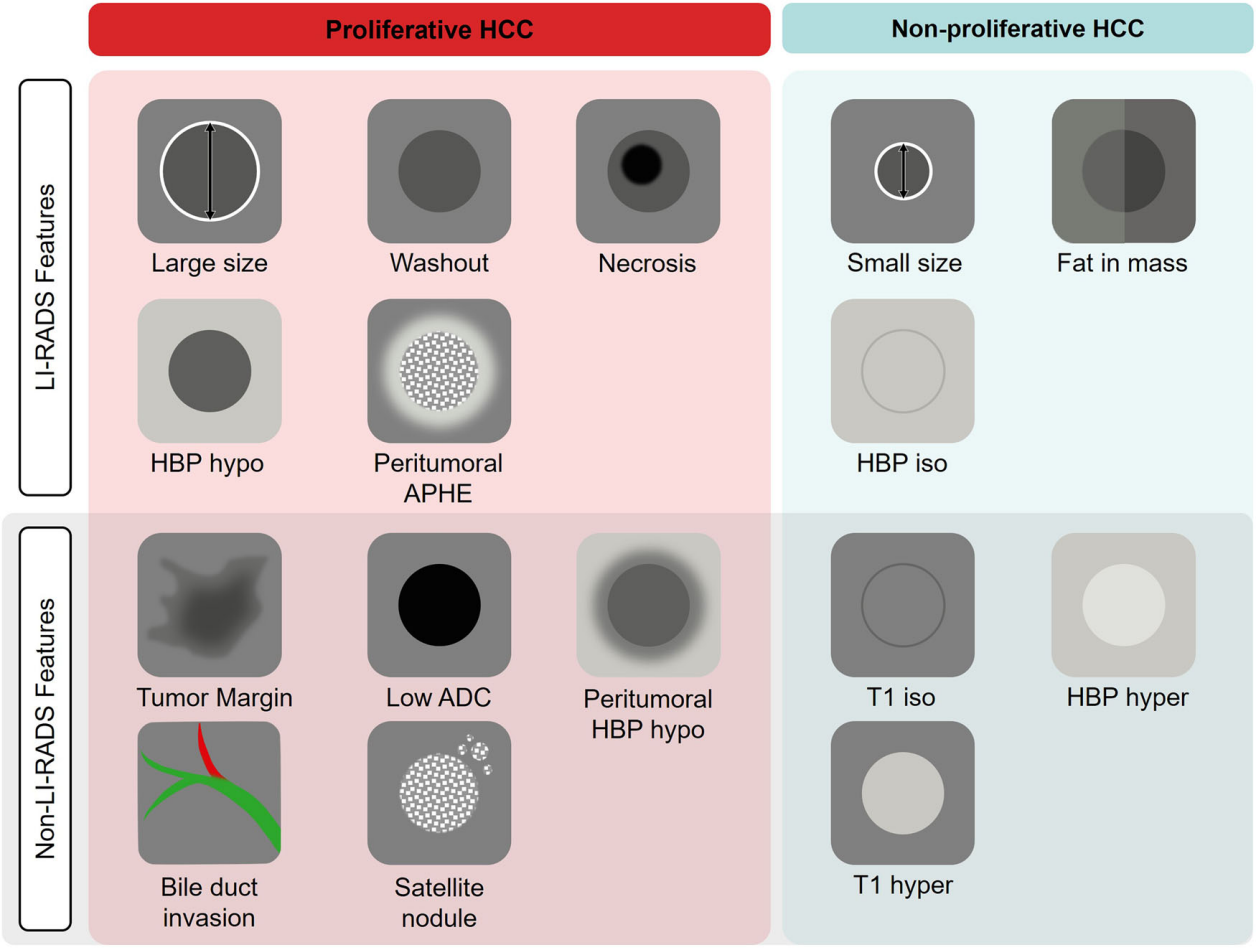
Prognostic imaging markers of HCC included as major or ancillary features in LI-RADS 2018 and those not currently included. ADC, apparent diffusion coefficient; APHE, arterial phase hyperenhancement; HBP, hepatobiliary phase

The LI-RADS promotes standardization of HCC imaging, including technique, diagnostic interpretation, lexicon, reporting, and management guidance. Certain features used in the diagnosis of HCC correlate with prognosis, as many features that increase the probability of HCC also may indicate a more aggressive cancer with worse outcomes. Still, there are emerging features that reflect poor prognosis that are not currently employed by LI-RADS. Figure 3 summarizes prognostic imaging features, indicating those that are integrated as major or ancillary imaging features in LI-RADS version 2018 and those that are associated with proliferative or non-proliferative HCCs.

The following section will outline each prognostic imaging feature. Figures 4–6 illustrate each prognostic feature. Case examples integrating different prognostic features can be found in Figs. 7–11.

**Fig. 4:**
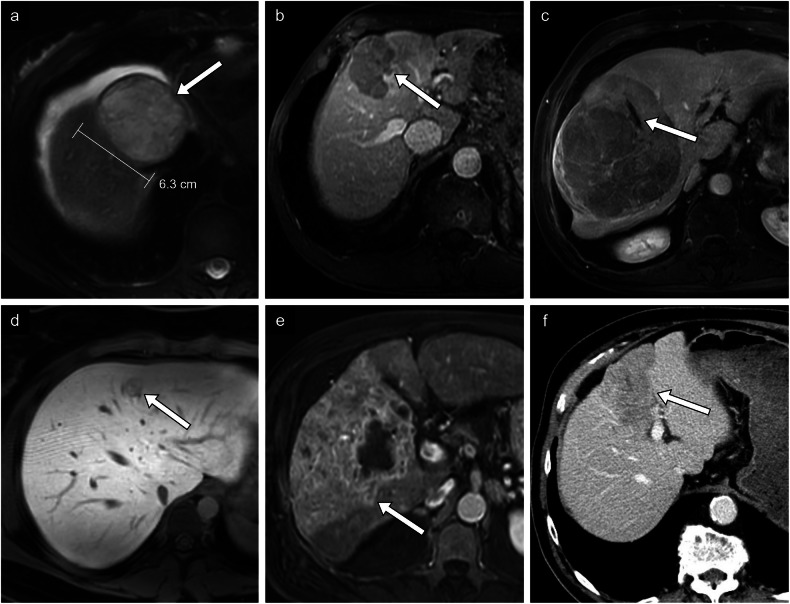
Proliferative prognostic imaging features. **a** HCC in a 50-year-old woman with autoimmune hepatitis. Axial T2-weighted fat-saturated MR image of a large 6.3-cm mass found in segment VIII. No histologic diagnosis was available. **b** HCC in a 75-year-old man with chronic hepatitis B viral infection without cirrhosis. Axial T1-weighted portal phase MR image of a mass with nonperipheral washout in segment VIII. Histologic diagnosis was a moderately differentiated HCC without microvascular invasion. **c** HCC in a 72-year-old male patient with chronic hepatitis B viral infection without cirrhosis. Axial T1-weighted arterial phase MR image of non-enhancing necrotic components within a large solid mass in segments V, VI, VII, and VIII. Histologic diagnosis confirmed a moderately differentiated HCC with microvascular invasion. **d** A 20-year-old woman with metabolic dysfunction-associated steatotic liver disease. On the T1-weighted hepatobiliary phase, the lesion is mildly hypointense compared to the surrounding liver parenchyma. Histologic diagnosis was an atypical hepatocellular neoplasm. **e** HCC in a 62-year-old man with alcohol-associated liver disease. Axial T1-weighted arterial phase MR image of multicentric hypointense lesions within the right liver with enhancement of the surrounding parenchyma. No histologic diagnosis was available. **f** HCC in an 83-year-old man with metabolic alcohol-associated liver disease. Axial portal phase CT image of a large multinodular infiltrative 9.0-cm mass found in segment IV. Histologic diagnosis was a poorly differentiated hepatocellular carcinoma

**Fig. 5:**
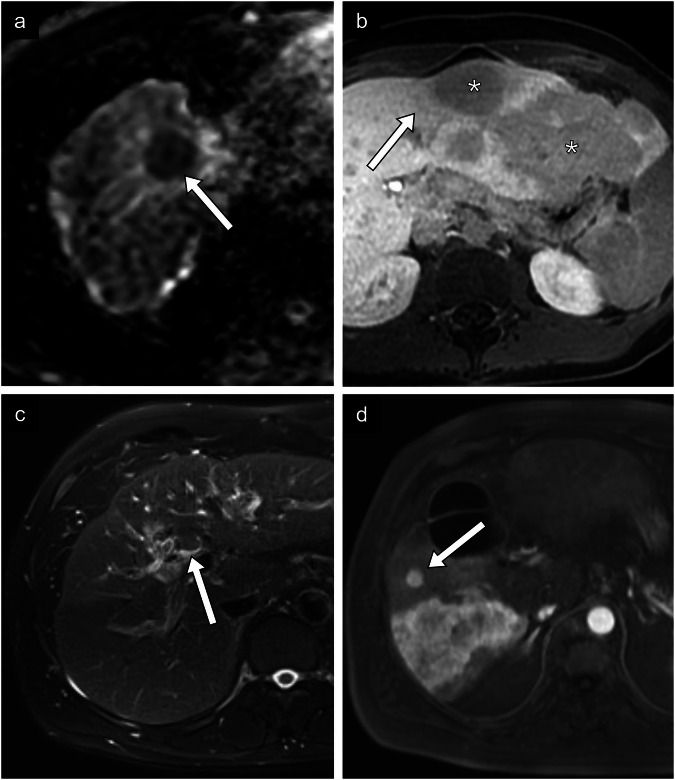
Proliferative prognostic imaging features (continued). **a** HCC in a 75-year-old woman with metabolic dysfunction-associated steatotic liver disease. ADC map shows a lesion in segment VIII with low signal intensity compared to the high signal intensity on diffusion-weighted imaging at *b* = 800 s/mm^2^ (not imaged). **b** HCC in a 24-year-old woman with a diffusely steatotic liver without fibrosis. Axial T1-weighted hepatobiliary phase MR image shows a 20-cm multilobular hypointense mass (*) with wedge-shaped mildly hypointense regions surrounding the mass. Histologic diagnosis was a moderately differentiated HCC of macrotrabecular massive type with fibrolamellar components. **c** HCC in a 58-year-old man with resolved hepatitis C viral infection and alcohol-associated liver disease. Axial T2-weighted fat-saturated MR image of a solid hyperintense mass at the common hepatic duct extending into the intrahepatic bile ducts. Histologic diagnosis was a well-differentiated HCC. **d** HCC in a 72-year-old man with metabolic and alcohol-related/associated liver disease. Axial T1-weighted arterial phase MR image of a large infiltrative mass found in segments VI and VII, with a small nodule measuring 1.5 cm situated close to the primary tumor corresponding to a satellite nodule. No histologic diagnosis was available

**Fig. 6:**
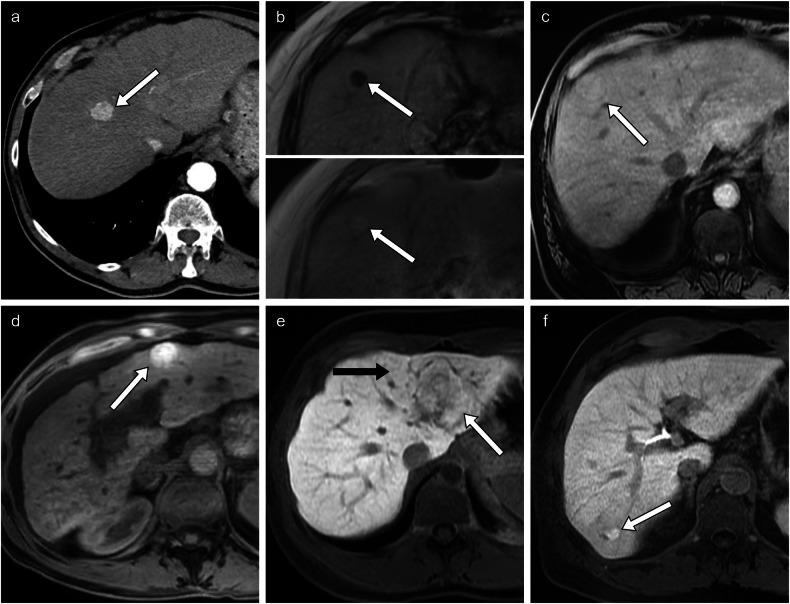
Non-proliferative prognostic imaging features. **a** HCC in a 73-year-old man with alcohol-associated liver disease. Axial arterial phase CT image of a small 1.8-cm lesion found in segment VIII. Histologic diagnosis was a well-differentiated HCC. **b** HCC in a 72-year-old man with alcohol-associated liver disease. Axial T1-weighted out-of-phase (top panel) and in-phase (bottom panel) MR images of a 1.4-cm nodule demonstrating signal loss in the out-of-phase image compatible with intralesional fat. Histologic diagnosis was a moderately differentiated steatohepatitic type HCC. **c** HCC in a 75-year-old man with alcohol-associated liver disease. Axial non-contrast T1-weighted MR image of an isointense lesion compared to the hepatic parenchyma in segment VIII. Histologic diagnosis was a well-differentiated HCC. **d** HCC in a 48-year-old woman with autoimmune cirrhosis. Axial non-contrast T1-weighted MR image of a hyperintense lesion compared to the hepatic parenchyma. No histologic diagnosis was available. **e** HCC in a 23-year-old woman without underlying chronic liver disease. Axial T1-weighted hepatobiliary phase MR image of 5.2-cm lesion in segment II demonstrating isointensity (white arrow) compared to the surrounding liver (black arrow). Histologic diagnosis is a well-differentiated HCC. **f** HCC in a 63-year-old man with chronic hepatitis C viral infection and alcohol-associated liver disease. Axial T1-weighted hepatobiliary phase MR image of a 1.5-cm hyperintense lesion in segment VI. Histologic diagnosis confirmed a moderately to poorly differentiated HCC

**Fig. 7:**
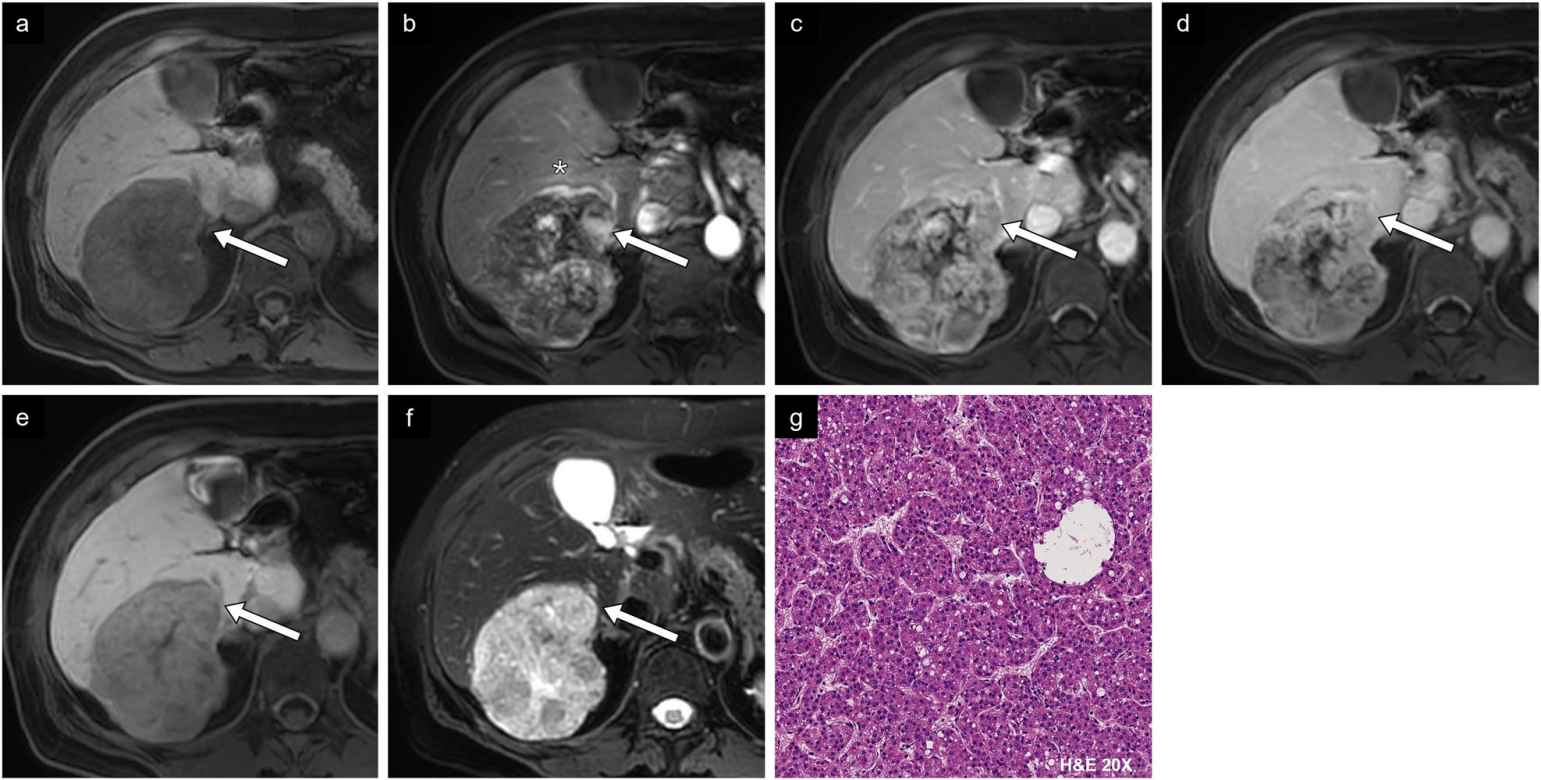
Case example of proliferative imaging features. HCC in a 57-year-old woman without underlying chronic liver disease. **a** Non-contrast T1-weighted image shows a hypointense, large, heterogeneous 7.5-cm mass in segment VI. **b** Arterial phase image with gadobenate dimeglumine shows tumoral and peritumoral hyperenhancement (*). **c** Portal and (**d**) delayed phase images show washout within the lesion. **e** On hepatobiliary phase, the lesion demonstrates low signal. **f** T2-weighted image shows marked hyperintensity. **g** Histologic diagnosis was a moderately differentiated trabecular-type HCC (conventional type). Note the prior left hepatectomy. H&E, hematoxylin and eosin

**Fig. 8:**
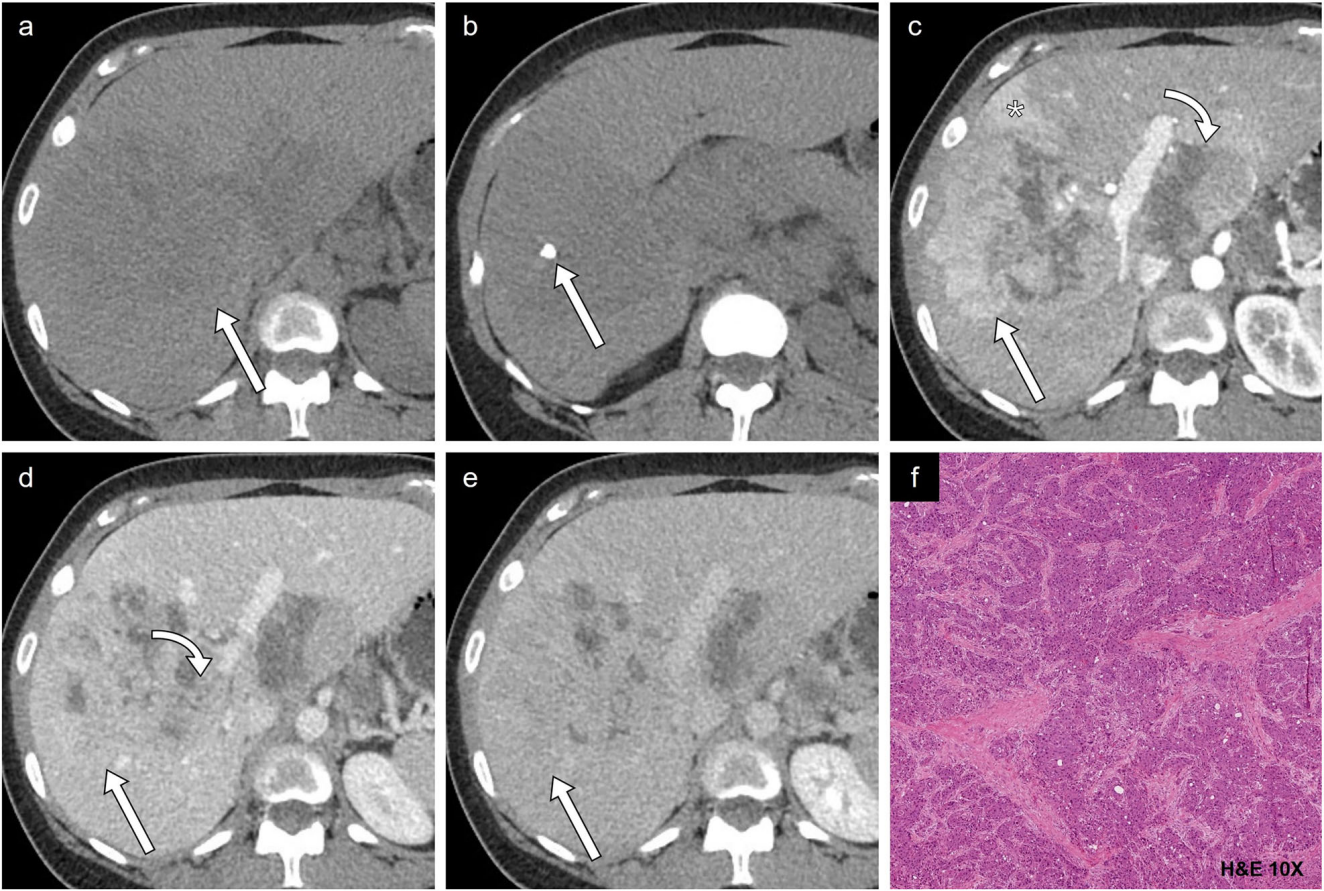
Case example of proliferative imaging features. HCC in a 34-year-old woman without underlying chronic liver disease. **a** Axial non-contrast CT image of an isodense large mass measuring 11.1 cm involving all segments in the right liver, and (**b**) a more inferior slice showing an internal calcification. **c** Arterial phase image shows hyperenhancement (straight arrow) with non-enhancing necrotic components (curved arrow) and peritumoral hyperenhancement (*). **d** Portal phase image shows nonperipheral washout (straight arrow) and right portal vein thrombosis (curved arrow). **e** Delayed phase image demonstrates nonperipheral washout. **f** Histologic examination showed a moderately differentiated HCC of fibrolamellar type. H&E, hematoxylin and eosin

**Fig. 9:**
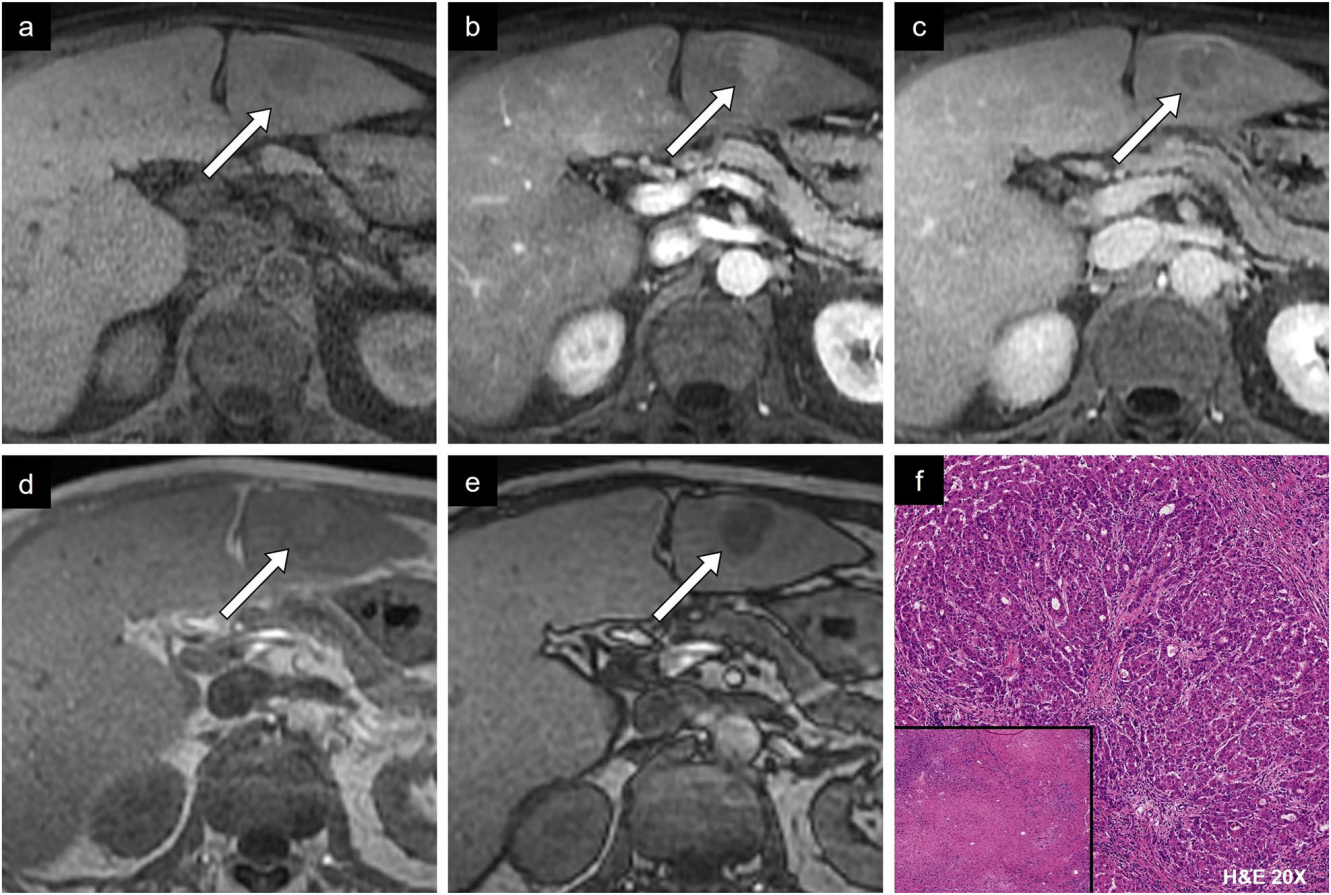
Case example of non-proliferative imaging features. HCC in a 58-year-old man with alcohol-associated liver disease. **a** Axial non-contrast T1-weighted MR image shows isointensity compared to the surrounding liver parenchyma. **b** Arterial phase image shows enhancement with (**c**) portal phase washout. **d** Axial in-phase and (**e**) out-of-phase images show a drop in intensity in out-of-phase imaging representing fat content. **f** Histologic diagnosis was a well-differentiated HCC. Two other fibrotic HCC lesions were identified, demonstrating complete treatment response (bottom left). H&E, hematoxylin and eosin

**Fig. 10:**
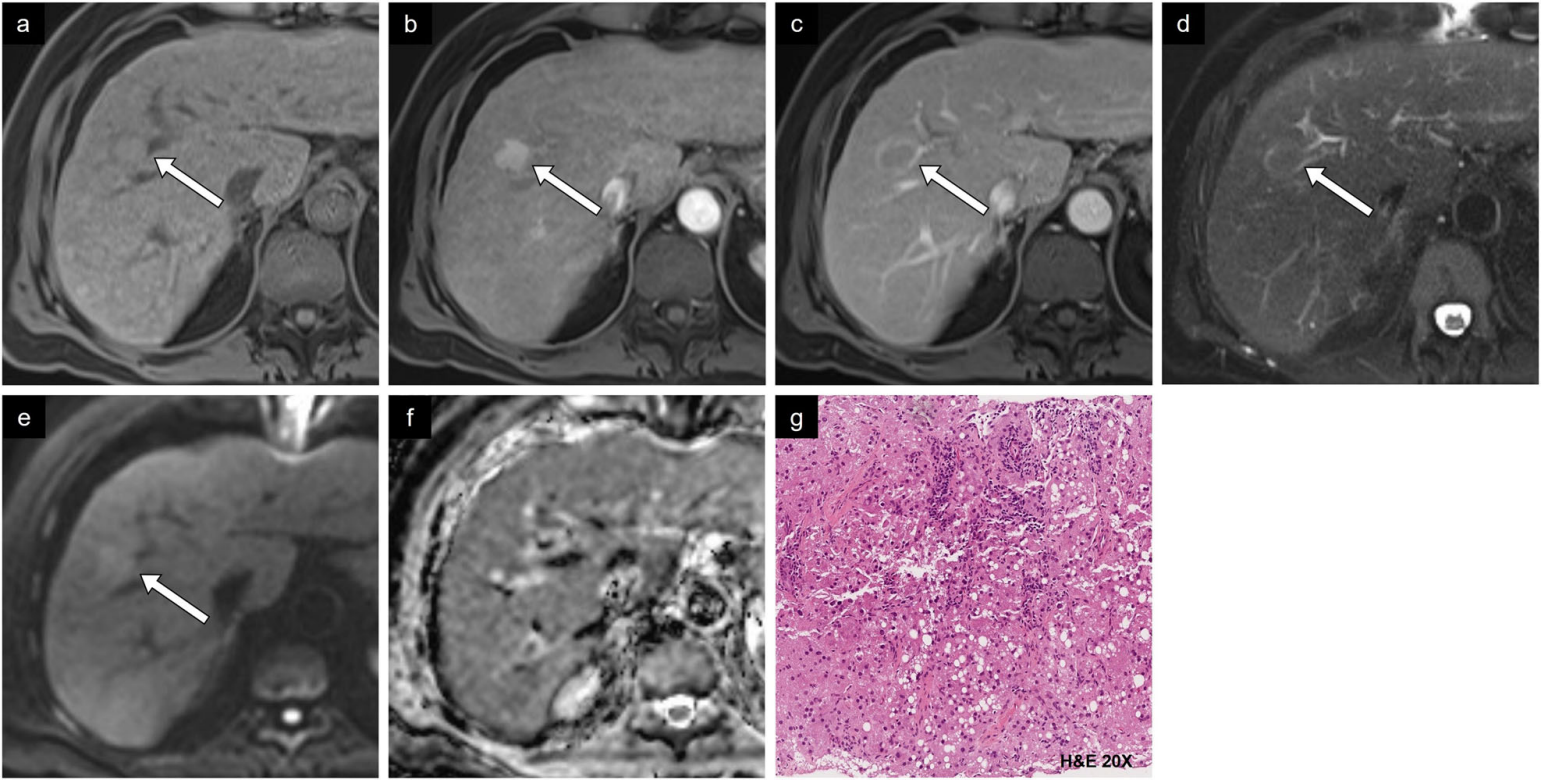
Case example of non-proliferative imaging features. HCC in a 74-year-old man with metabolic dysfunction-associated steatotic liver disease. **a** Non-contrast T1-weighted image shows an isointense 2.2-cm lesion in segment VIII. **b** Arterial phase image shows irregular tumor margin with hyperenhancement and **c** washout on portal phase image. **d** T2-weighted image shows a similar signal to liver parenchyma. **e** Diffusion-weighted imaging and **f** ADC map show absence of diffusion restriction. **g** Histologic diagnosis was a steatohepatitic type HCC. H&E, hematoxylin and eosin

**Fig. 11:**
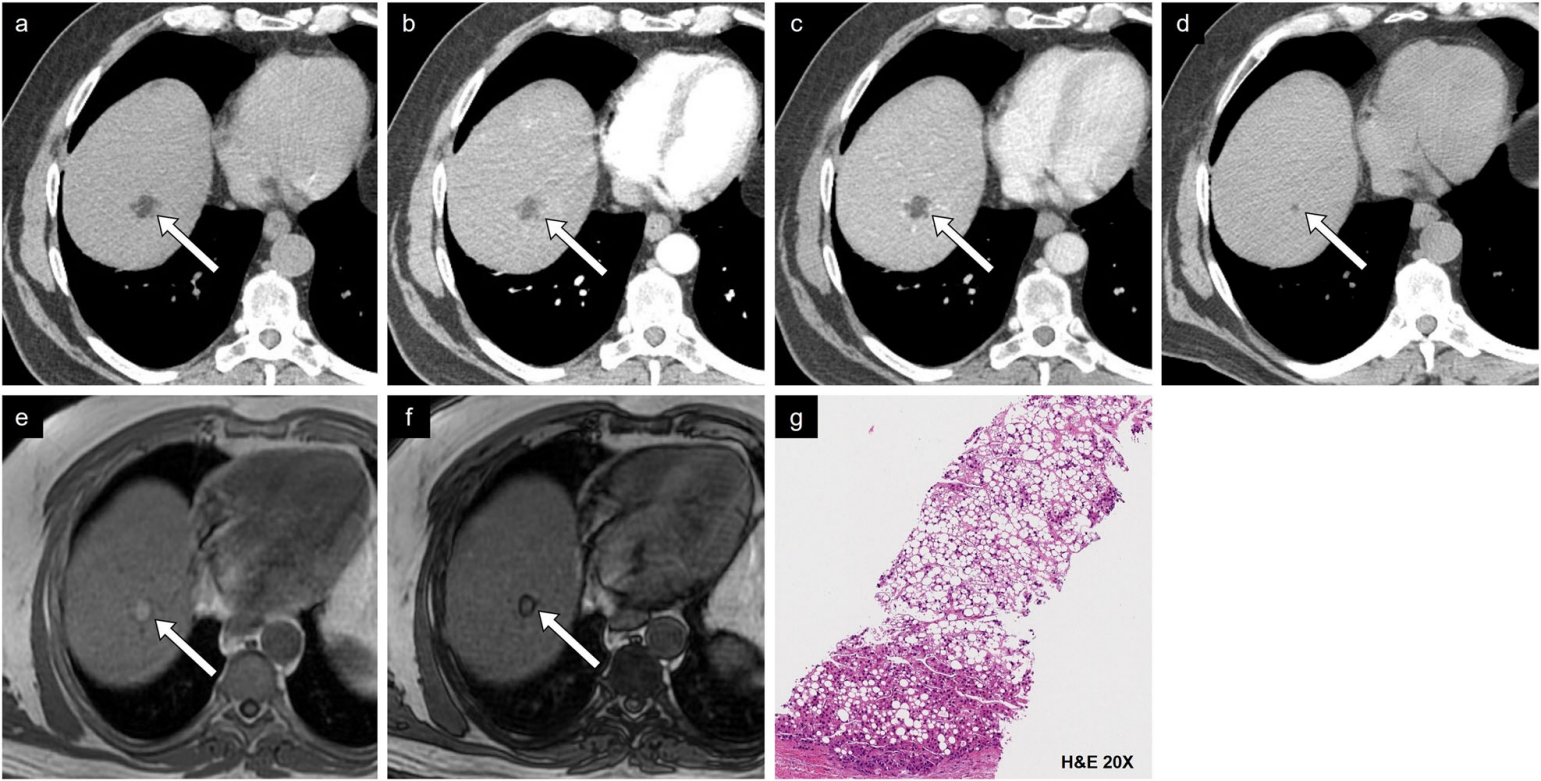
Case example of non-proliferative imaging features. HCC in a 73-year-old man with metabolic dysfunction-associated steatotic liver disease. **a** Non-contrast CT image shows a small lesion measuring 2.3 cm between segments VII and VIII with density compatible with macroscopic intralesional fat. **b** Arterial phase and (**c**) portal phase images show a hypodense lesion. **d** On CT performed 2 years prior, the lesion measured 0.6 cm on the non-contrast phase, corresponding to threshold growth. **e** In-phase and (**f**) out-of-phase MR images show a drop in signal in out-of-phase imaging. **g** Histologic examination showed a well-differentiated HCC of steatohepatitic type. H&E, hematoxylin and eosin

### Proliferative features

#### Large size

Tumor growth is linked to immune modulation, which produces cytokines and growth factors, such as vascular endothelial growth factor (VEGF), and concomitantly suppresses anti-tumor properties of surrounding lymphocytes [20]. Chronic inflammation plays a complex role in tumorigenesis, with both inflammation within fibrotic parenchyma and tumor-associated inflammation contributing to a microenvironment conducive to tumoral proliferation. Poor cellular differentiation and elevated AFP have been associated with large tumor size [21]. Observation size, measured as the largest outer-to-outer edge dimension, is among the major features in LI-RADS 2018 diagnostic criteria, with a size of 2 cm or greater increasing the probability of malignancy [4]. Tumors larger than 5 cm have been associated with poorly differentiated HCC, advanced stage, and worse survival outcomes (Fig. 4a) [22, 23]. Tumor size determines the T stage in the current TNM staging of HCC according to the American Joint Committee on Cancer 8th edition [14].

#### Washout

Tumor washout is defined as a reduction of enhancement from earlier to later phase, resulting in hypoenhancement relative to the liver. Washout is assessed on portal venous phase or delayed phase imaging with extracellular agents (ECA) or gadobenate dimeglumine, and on portal venous phase only with gadoxetate disodium (Fig. 4b). While nonperipheral washout constitutes a major LI-RADS imaging feature, it may also indicate increased tumor aggressiveness. Washout reflects a combination of concomitant pathological processes, including reduced portal venous supply and compensatory increased arterial blood supply within the tumor, retention of contrast in surrounding fibrotic parenchyma, and early venous drainage of intratumoral contrast [24]. Shorter washout times have been associated with poorly differentiated HCC [25, 26].

#### Necrosis in solid mass

Necrosis can be identified by a non-enhancing area within a solid mass, not attributable to a cystic component, prior treatment or intralesional hemorrhage (Fig. 4c). It is considered a nontargetoid LR-M (probably or definitely malignant, not HCC-specific) feature in the LI-RADS v2018. Tumor necrosis reflects intratumoral hypoxia in the context of rapid growth of a mass, suggestive of more aggressive cancer. Necrotic tumor also releases pro-inflammatory molecules such as interleukin-33 or HMGB1, which may foster tumor growth [27]. A 2024 meta-analysis of ten studies showed that necrosis was associated with the macrotrabecular massive subtype of HCC, which has a tendency for metastasis and disease recurrence [28].

#### HBP hypointensity

Hepatobiliary-specific contrast agents have a unique pharmacodynamic profile involving uptake by hepatocytes using transporters such as organic anion transporting polypeptide (OATP) 1B3. Contrast is then excreted into the biliary system by multidrug resistance-associated protein (MRP) 2, expressed on the canalicular side of hepatocytes [29]. The level of expression of transporters and proteins involved in the pharmacodynamics of hepatobiliary-specific contrast agents determines the degree of hepatobiliary phase (HBP) enhancement. HBP hypointensity is defined as signal intensity lower than the surrounding liver (considered mild if higher than vessels but marked if similar to or lower than vessels) and is a current LI-RADS ancillary feature favoring malignancy in general (Fig. 4d). Low HBP enhancement has been associated with a reduced expression of OATP and poorer cellular differentiation [30].

#### Peritumoral AP hyperenhancement

LI-RADS defines peritumoral arterial phase (AP) hyperenhancement as a nonmasslike area adjacent to a mass with hyperenhancement in AP and fade in the post-AP phases. This feature can be seen in early AP or the late AP/early portal venous phase (synonymous with corona enhancement) (Fig. 4e). This also represents an ancillary feature favoring malignancy in general. The pathophysiology of peritumoral AP hyperenhancement is unclear and likely multifactorial. One proposed mechanism is arterioportal shunting caused by obstruction of venous and portal venules in surrounding parenchyma due to infiltrating tumor or thrombi of microvessels (i.e., microvascular invasion) [5]. Corona enhancement has been linked with increased recurrence and high-grade HCC [31].

#### Tumor margin

Proliferation of tumor cells beyond the tumor capsule and into surrounding parenchyma or into minute vessels may translate into a nonsmooth tumor margin. Moreover, nonsmooth margins possibly reflect the inherently more aggressive and histologically high-grade progenitor phenotype of HCC, which is associated with CK19 expression. Patterns of tumor margins associated with negative prognostic outcomes include nonsmooth, irregular, interrupted, presence of nodular projections (focal extranodular or multinodular), and infiltrative (Fig. 4f). Nonsmooth tumor margin has been associated with microvascular invasion (MVI), macrotrabecular massive histopathologic subtype, and tumor recurrence [32, 33].

#### Low ADC

High cellular content of a tumor restricts the diffusion of water molecules, resulting in a high diffusion-weighted imaging (DWI) signal and low apparent diffusion coefficient (ADC) (Fig. 5a). In hepatocarcinogenesis, low ADC is possibly a result of increased cellular atypia, such as high mitosis and high nucleus/cytoplasm ratio [34]. Low signal intensity compared to the surrounding liver on ADC maps (*b* = 800 s/mm^2^) has been reported as an independent predictive feature for poor cellular differentiation, microscopic portal vein invasion, and higher disease recurrence after hepatectomy, although it is not considered a LI-RADS feature yet [35, 36]. The corollary is also true: less aggressive HCC has been associated with higher ADC [24].

#### Peritumoral HBP hypointensity

Peritumoral HBP hypointensity is defined as nonmasslike hypointensity of liver adjacent to a mass in HBP (Fig. 5b). Hepatocyte transporters responsible for hepatobiliary-specific contrast uptake and excretion, may not only be impaired within the primary tumor (as with HBP hypointense lesions), but also might be involved in the surrounding parenchyma via local tumoral invasion within portal venules [5]. Similar to tumoral HBP hypointensity, peritumoral HBP hypointensity has been associated with microvascular invasion [37]. Peritumoral hypointensity in HBP has also been independently associated with early recurrence after different therapies [38, 39]. Kim et al demonstrated that peritumoral HBP hypointensity was even linked with greater recurrence rates after liver transplantation with an adjusted hazard ratio of 4.24 [40].

#### Bile duct invasion

Advanced HCC may invade intra- or extrahepatic bile ducts and induce various complications with a direct impact on prognosis (Fig. 5c). Bile duct tumor invasion frequently presents with hyperbilirubinemia, which increases the risk of post-hepatectomy liver failure, biliary sepsis and intra-operative bleeding [41]. Moreover, concomitant microvascular invasion is commonly found among patients with bile duct invasion [42]. Some suggest that the intrinsic infiltrative nature of HCC with bile duct invasion influences prognosis, although this remains controversial [42]. There is a paucity of clinical data available on bile duct-invasive HCC; however, prognosis is generally considered poor, with high rates of recurrence after surgery and reduced overall survival [42, 43].

#### Satellite nodule

Satellite lesions are defined as small tumor nodules measuring up to 2 cm within close proximity (< 2 cm) of the main tumor; however, they are not yet part of the LI-RADS lexicon (Fig. 5d). Satellite nodules represent metastatic deposits within the surrounding hepatic parenchyma secondary to contiguous spread or hematogenous dissemination of tumor cells. Some studies have shown that satellite nodules possess a particular genetic profile and often express genes associated with tumor metastasis, like CHML and IMP3 [44, 45]. A 2020 study showed that satellite nodules are associated with recurrence of LR-5 (definite HCC) observations after liver resection and may be a useful marker to stratify patients preoperatively [38].

### Non-proliferative features

#### Small size

Tumors less than 2 cm in size are generally less aggressive than large tumors (Fig. 6a). Smaller tumor size indicates less rapid tumoral cell replication, less apoptotic resistance, and more often well-differentiated tumors without microvascular invasion [5, 46]. Small tumor size is the single most important prognostic factor detectable with imaging. In a recent study including over 14,000 participants with HCC, those with tumors less than 2 cm who underwent surgery or surgery plus chemotherapy had longer survival than those with larger tumors [47].

#### Fat in mass

Fat content is associated with early and non-metastatic HCC or with the steatohepatitic subtype, which generally has a more favorable prognosis. Fat in mass represents an ancillary LI-RADS feature that favors HCC. On MRI, fat in mass is represented by a drop in intensity between in- and opposed-phase images (Fig. 6b). A large multicenter study found that a homogeneous pattern of fat in mass was associated with longer recurrence-free survival and overall survival after resection [48].

#### T1 iso- and hyperintensity

Signal intensity on unenhanced T1-weighted images has been associated with good histologic differentiation grade, which is possibly attributable to metal accumulation, such as copper, and occasionally microscopic fat, within the tumor [49]. Hyperintense lesions in T1 have been associated with earlier-stage HCC and well-differentiated tumor cells [49]. Hyper- and isointense lesions generally have more favorable histologic differentiation than hypointense lesions (Fig. 6c, d) [50].

#### HBP iso- and hyperenhancement

As opposed to HBP hypointensity, HBP iso- and hyperintensity are correlated with elevated OATP expression by tumor cells (Fig. 6e, f). Overexpression of OATP has been associated with the Wnt-β-catenin signaling pathway, which is linked with well-differentiated HCC and aligns with the non-proliferative S3 class [7, 51]. Ueno et al found that tumor enhancement in HBP had a sensitivity of 78.9% and a specificity of 81.7%, for Wnt-β-catenin activated HCC [52]. Increased MRP3 expression, the principal export transporter of hepatobiliary-specific contrast agents, may also play a role in HBP signal, possibly as a response to increased OATP expression [53]. Importantly, intralesional hyperintensity on HBP has been associated with decreased disease recurrence after surgery [53]. Choi et al have reported a longer time to tumor recurrence after surgery in iso- to hyperintense lesions compared with hypointense observations on HBP [54].

### Microvascular invasion

**Fig. 12:**
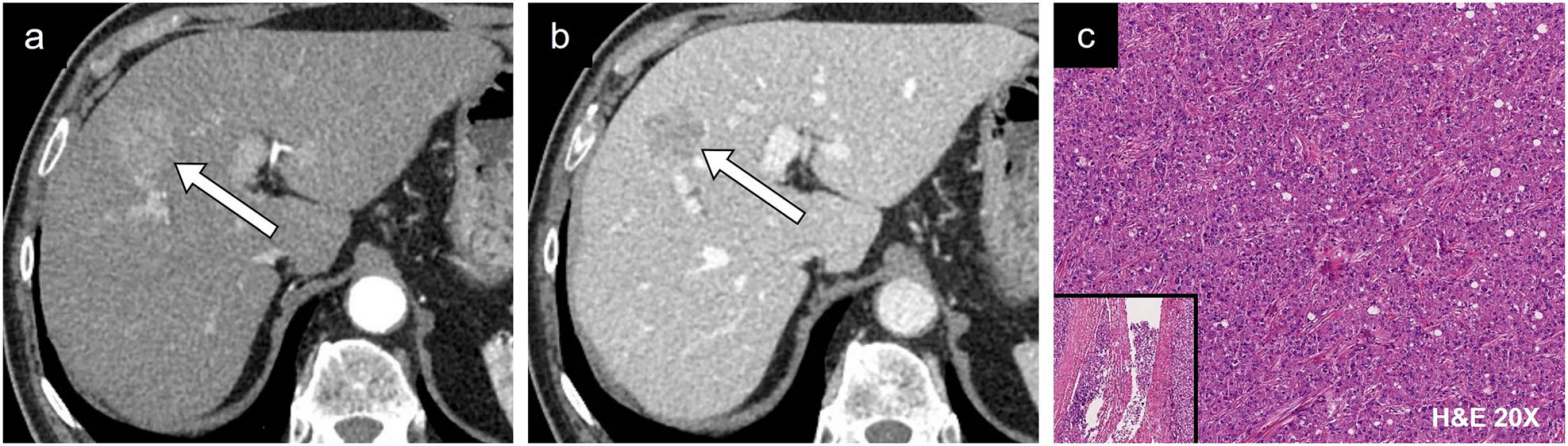
Prognostic imaging markers of microvascular invasion. HCC in a 74-year-old man with chronic hepatitis C viral infection. **a** Axial arterial phase CT image showing an ill-defined hyperenhancing lesion at the junction of segment VIII and IV. **b** Portal phase image shows washout. **c** On histology, the lesion was a moderately differentiated HCC of trabecular-solid type (conventional type) with portal vein invasion (bottom left) and lymphatic invasion. There was no biliary or neural involvement. H&E, hematoxylin and eosin

Microvascular invasion (MVI) is an independent prognostic marker that influences disease-free survival and overall survival [55]. Early spread of cancer cells into the bloodstream is a key mechanism for developing disease recurrence and metastasis. The infiltrative nature within the surrounding parenchyma and vascular structures has a direct impact on prognosis. However, confirmation of microvascular invasion requires histopathological diagnosis using resection or biopsy. Numerous imaging features have shown promise for detecting MVI noninvasively. Figure 12 shows different radiological features of microvascular invasion and the correlation with histology.

Several studies show that tumor size greater than 5 cm is a reliable predictive feature for MVI [56–58]. Multiple foci of HCC within the liver may be predictive of MVI as multifocality suggests direct tumoral spread. It may also reflect tumor propagation via the bloodstream to produce additional liver implants.

A nonsmooth margin is a strong predictor for MVI. Tumors with irregular borders and parenchymal infiltration at imaging are often linked with tumor in the vein and extrahepatic metastasis [59]. Diffusion-weighted imaging may be helpful for the evaluation of early MVI, as the ADC value has been directly correlated with MVI. A threshold of 1.11 × 10^−3^ mm^2^/s, or lower, was shown in one study to have a sensitivity of 93.5% and specificity of 72.2% for MVI [60]. One hypothesis is that HCC with MVI may be more hypercellular than HCC without MVI. Another explanation may be a decrease in microcapillary profusion, demonstrated by low ADC.

Peritumoral AP hyperenhancement is a highly specific finding for MVI, albeit not sensitive. A meta-analysis found that the specificity of peritumoral AP hyperenhancement for MVI was about 90% while the sensitivity was only 29% [61]. In the same study, peritumoral HBP hypoenhancement yielded a sensitivity of 72% and a high specificity of up to 94% for MVI.

### Predictors of treatment responsiveness

Novel therapeutic modalities, particularly systemic therapies, have emerged for HCC in the past decade, with patient selection algorithms such as the Barcelona Clinic Liver Cancer (BCLC) staging system. These have helped improve patient outcomes and increase overall survival [18]. According to the BCLC algorithm, early-stage HCC is ideally managed with surgical resection, transplantation or ablative therapy, while intermediate-stage HCC is recommended to be treated with transarterial therapy, such as transarterial chemoembolization (TACE). Systemic therapy is the primary therapeutic modality for advanced-stage HCC [17]. Besides the advancements in treatment options, poor survival rates are still documented for metastatic HCC [62]. Stratifying the pattern of disease progression early through the identification of prognostic and predictive features is key to optimizing treatment selection [17]. Predictive imaging shows promise for this purpose (Tables 3, 4).

**Table 3:** Predictive imaging markers of surgical resection and transplantation

Treatment	Reference	Study design	No. of patients	Follow-up duration	Poor predictive imaging finding	Primary outcome
Surgical resection	Shin et al [63]	Retrospective, single-center	281	2 years	LR-M compared to LR-4/5, largest tumor size of ≥ 3 cm	Early recurrence
Surgical resection	Jiang et al [65]	Retrospective, single-center	532	1 to 11 years	Tumor size, arterial phase hyperenhancement	HCC recurrence
Surgical resection	Wei et al [38]	Retrospective, single-center	103	Median of 22.1 months	Corona enhancement, peritumoral hypointensity on hepatobiliary phase, satellite nodule	Early recurrence
Surgical resection	Wei et al [31]	Retrospective, single-center	111	Median of 22.1 months	Blood products in mass, corona enhancement	Recurrence
Surgical resection	Zhang et al [67]	Retrospective, single-center	82	1 year	Corona enhancement, irregular tumor margin	Early recurrence
Surgical resection	Chen et al [68]	Retrospective, single-center	149	3 years	Multifocal tumors, absence of fat in mass, nonsmooth tumor margin	Recurrence
Surgical resection	Bae et al [39]	Retrospective, single-center	183	Median of 51 months	Large tumor size on hepatobiliary phase	Disease-free survival, overall survival
Liver transplant	Lee et al [64]	Retrospective, multicenter	140	7 years	LR-M compared to LR-4/5	Recurrence-free survival

**Table 4:** Predictive imaging markers of radiofrequency ablation (RFA) and transarterial chemoembolization (TACE)

Treatment	Reference	Study design	No. of patients	Follow-up duration	Poor predictive imaging finding	Primary outcome
RFA	Iwamoto et al [71]	Retrospective, single-center	91	Median of 39.5 months	Non-hypervascular hypointense nodules on hepatobiliary phase	Intrahepatic distant recurrence
RFA	Petukhova-Greenstein et al [70]	Retrospective, single-center	65	2 years	Multifocality, continuity of an enhancing capsule, higher radiomic signature based on nodular and perinodular features	Progression-free survival
RFA	Bae et al [39]	Retrospective, single-center	183	Median of 51 months	Satellite nodules on hepatobiliary phase, peritumoral hypointensity on hepatobiliary phase	Disease-free survival, overall survival
RFA	Mori et al [72]	Retrospective, single-center	136	2 years	Hypointensity on apparent diffusion coefficient (ADC) map	Cumulative recurrence rates, cumulative survival rates
RFA	Wang et al [73]	Retrospective, single-center	115	Average of 377 days	Peritumoral hypointensity on hepatobiliary phase, irregular protruding margin on hepatobiliary phase	Local tumor progression, intrahepatic distant recurrence
RFA	Lee et al [74]	Retrospective, single-center	467	10 years	Periportal location, subphrenic location, size ≥ 1.5 to < 2.0 cm and ≥ 2.0 cm	Local tumor progression
TACE	Vesselle et al [75]	Prospective, single-center	172	1 month	Nodules located in segments I and IV	Complete response
TACE	Bryant et al [76]	Retrospective, single-center	115	75 days	Centrally located tumors (less than 4 cm from portal vein bifurcation)	Complete response > 90% tumor necrosis
TACE	Kim et al [77]	Retrospective, single-center	88	Median 28.5 months	Gross vascular invasion, bile duct invasion, irregular tumor margin, peripheral ragged enhancement, satellite nodules	Non-response (stable disease or progression), overall survival
TACE	Nakano et al [78]	Retrospective, single-center	35	Median 1.6 months	Large tumors, contact with liver surface, residual supplying vessels	Local recurrence
TACE	Ishimaru et al [79]	Retrospective, single-center	60	3 years	Low uptake of gadoxetic acid	Local recurrence

Numerous LI-RADS features were reported to hold predictive value for patients undergoing therapeutic interventions. LR-M category HCCs, compared to LR-4 or LR-5, were observed to have a higher likelihood of early recurrence following surgical resection and liver transplantation [63, 64]. Rim arterial phase hyperenhancement, one of the targetoid LR-M features, was also correlated with advanced-stage HCC recurrence [65]. In a recent retrospective study, LR-M early-stage HCCs exhibited a higher growth rate than LR-3, LR-4, and LR-5 HCCs, supporting the category-specific increased risk of recurrence [66]. Some of the ancillary features were also associated with an increased risk of tumor recurrence in patients undergoing partial hepatectomy for HCC. The predictive markers of recurrence consisted of blood products in mass, corona enhancement, and the absence of fat in mass [31, 38, 67, 68]. A key ancillary feature, HBP hypointensity, has been investigated as a predictive marker for different treatment modalities. A meta-analysis of five studies concluded that pretreatment HBP hypointense HCC had a higher risk of recurrence following surgical resection or transplantation [69]. Nonsmooth tumor margin, multifocality, and satellite nodules were non-LI-RADS features that were predictors of poor outcomes following curative therapies [38, 67, 68].

Non-hypervascular hypointense nodules, satellite nodules, and multifocality were likewise predictive markers of recurrence after radiofrequency ablation (RFA) [39, 70, 71]. Hepatobiliary phase peritumoral hypointensity of nodules and low ADC were additional markers of recurrence and poorer overall survival for patients who underwent RFA [39, 72, 73]. The location of HCC was also relevant for predicting RFA response, with periportal and subphrenic locations associated with posttreatment progression [74].

Some studies have evaluated the efficacy of TACE and its predictive markers of complete response. A lower complete response to TACE was associated with tumors located in the 1st and 4th Couinaud’s segments or less than four centimeters away from the portal vein bifurcation [75, 76]. Tumors with irregular margins, gross vascular invasion, bile duct invasion, and peripheral ragged enhancement or satellite nodules remained stable or progressed following TACE more frequently [77]. Nakano et al demonstrated that large tumor size and contact with the liver surface of the nodule increased the risk of local recurrence following drug-eluting bead-TACE [78]. HBP hypointensity was also a predictive marker associated with suboptimal outcomes after TACE [79].

Immune checkpoint inhibitors and targeted therapies are the primary treatment options for patients with advanced-stage HCC. These treatments are actively expanding, and the main inhibitors recommended in the guidelines are programmed cell death protein 1 (PD1) or PD-L1 inhibitors, cytotoxic T-lymphocyte-associated protein 4 (CTLA-4) inhibitors, VEGF inhibitors, and multi-kinase inhibitors [80]. Since treatment response largely depends on the tumor landscape and tumor immune microenvironment characteristics, imaging markers that can stratify subtypes of HCC based on these features would be an essential cornerstone in optimizing patient treatment and maximizing prognosis. In a study conducted by Sun et al, irregular tumor margins and peritumoral low signal intensity on the HBP phase of MRI were associated with higher tumor-infiltrating CD8+ cell density and PD-L1 expression [81]. Other markers, such as steatotic HCCs, were more susceptible to the anti-PD-L1 and anti-VEGF combination, resulting in longer progression-free survival [82]. In contrast, heterogeneous signal intensity and HBP hyperintensity were associated with decreased progression-free survival in patients who underwent PD-1 monotherapy or a combination of PD-1 inhibitors and VEGF inhibitors in multiple studies [83–85].

Radiomics refers to the extraction of quantitative data from digital medical images, including texture, size, shape, and transformation-based features [86]. When combined with machine learning, radiomics can permit the identification of imaging features associated with clinical outcomes [87]. For instance, Peng et al used pre-TACE CT imaging of HCC patients and applied radiomics with machine learning to develop a predictive model for TACE response. The model had a high predictive accuracy with an area under the receiver operating characteristic curve of 91.7% for selecting patients that would benefit from this treatment modality, and these patients had a longer progression-free survival and overall survival than the ones that were not selected by this model and underwent TACE [88]. Various other models have been studied for their predictive performance in optimizing patient selection for the above therapies [89, 90]. While this is a promising field that could improve HCC outcomes, the inadequate generalizability of the models, the need for standardized protocols to assure homogeneity between studies, and the absence of clinical validation are some challenges that impede its complete integration into clinical practice.

### Cost-effectiveness

There are remaining gaps that will need to be addressed before imaging-based prognostication and prediction are implemented clinically. For one, it must be determined whether imaging-based models have sufficiently high positive and negative predictive values to guide management decisions reliably. Second, the cost-effectiveness of the models should be compared to alternative approaches such as tissue sampling with molecular characterization or liquid biopsy. With regard to imaging, the cost of the imaging exam itself is a non-issue as the imaging is being performed routinely for other purposes, such as diagnosis, staging, monitoring, and/or treatment response. However, the cost of the prognostic/prediction models remains unclear. Should commercial models be developed and approved for clinical use, the financial burden of the algorithm must be assessed. Will the models be paid for by the patient, the insurance, the radiology department, or the health center? Moreover, the overall cost-effectiveness of integrating such models into standard clinical workflows requires careful evaluation.

### Limitations

There are limitations to the available literature on prognostic imaging features. First, the expanding role of imaging has introduced significant selection bias in tissue samples. The associations between prognostic imaging features and pathologic findings are made possible by obtaining tissue samples. As imaging has become increasingly accurate in HCC diagnosis, fewer patients undergo tissue sampling, and recent studies on pathology-proven HCC may disproportionately represent atypical cases, reducing generalizability.

Second, the specificity of many findings may be limited. Certain imaging findings correlated with a histologic and molecular subtype; however, they lacked specificity and could be found in other situations. For example, fat in mass was more frequently associated with steatohepatitic HCC, but it can also be an indicator of early-stage HCC [24].

Moreover, there are major differences in MR imaging practices across the world. For example, hepatobiliary-specific contrast agents are regularly utilized in Eastern populations, while validation of hepatobiliary contrast agents is needed in Western populations. Many determinants of prognosis remain understudied, such as geographic, ethnic, and socioeconomic disparities [91].

Differences in etiologies, genetic background, and access to treatment limit the generalizability of single-center retrospective studies. While LI-RADS provides minimum technical requirements for CT/MRI examinations, the imaging protocols are not standardized across institutions [92]. Hence, differences in choice of preferred imaging modality (CEUS, CT, or MRI), contrast injection protocols, image acquisition parameters, and reconstruction parameters may affect the external validity of predictive and prognostic markers in the literature [93]. A focus on standardizing reporting terms related to prognosis, as proposed by expert consensus, is crucial for facilitating further research on these markers and eventual implementation [94].

Overall, the prognostic and predictive features are not yet well defined. Most studies have primarily focused on patients with HBV living in Asian countries and have not yet been validated in prospective studies. Furthermore, it is not clear if any prognostic feature has sufficiently accurate diagnostic performance to direct clinical management.

## Future directions

Imaging has a central role in the diagnosis and characterization of HCC. By adopting the lexicon of prognostic features in conjunction with established LI-RADS terms, radiologists can communicate increasingly accurate information on clinical outcomes and stratify tumor aggressiveness. As emerging data support novel imaging markers in HCC assessment and prognostication, there is growing interest in adopting these imaging findings into the radiologist’s checklist. Proposed investigational templates can be found in the Electronic Supplementary Material, Appendix 1.

Prognostic and predictive features can potentially guide therapeutic selection by identifying the treatment(s) most likely to be efficacious. Further research into molecular data is essential to advancing targeted immunotherapy, which potentially represents the highest standard of individualized therapy [11].

Artificial intelligence models and radiomics can offer a quantitative assessment of prognosis and treatment response. The potential application of radiomics to this field of predictive and prognostic markers may enable the creation of precision-based medicine algorithms, allowing for the optimization of treatment plans and follow-ups with risk stratification. Radiomics-based models that integrate selected clinical features have demonstrated the ability to predict histologic and genetic characteristics, tumor recurrence, treatment response, and patient survival in HCC [86]. With further radiomics-based models being validated in independent cohorts, clinical application remains rare if not absent due to suboptimal generalizability, transparency, and relatively low levels of evidence. Future prospective studies with large populations and externally validated models will be required prior to clinical adoption.

## Conclusion

The role of imaging in the diagnosis and surveillance of HCC is becoming increasingly important. Several studies have gone beyond the diagnostic scope and investigated the prognostic and predictive value of CT and MRI. Predictive and prognostic features represent non-invasive tools that aid in evaluating tumor aggressiveness and personalizing HCC treatment plans. Continued correlation with imaging and genetic data is essential to better understand the many faces of HCC.

## Supplementary information

ELECTRONIC SUPPLEMENTARY MATERIAL

## Data Availability

The data that support the findings of this study are available from the corresponding author upon reasonable request.

## Abbreviations

ADC: Apparent diffusion coefficient
AFP: Alpha-fetoprotein
ALD: Alcohol-related liver disease
AP: Arterial phase
BCLC: Barcelona Clinic Liver Cancer
CT: Computed tomography
HBP: Hepatobiliary phase
HBV: Hepatitis B virus
HCC: Hepatocellular carcinoma
HCV: Hepatitis C virus
LI-RADS: Liver Imaging Reporting and Data System
MASLD: Metabolic dysfunction-associated steatotic liver disease
MRI: Magnetic resonance imaging
MRP: Multidrug resistance-associated protein
MVI: Microvascular invasion
NOS: Not otherwise specified
OATP: Organic anion transporting polypeptide
RFA: Radiofrequency ablation
TACE: Transarterial chemoembolization
VEGF: Vascular endothelial growth factor
WHO: World Health Organization
