# Supplementary material for: Prognostic and predictive imaging markers of hepatocellular carcinoma: a pictorial essay
Source: Insights Imaging. 2025 Aug 15;16:181. doi: 10.1186/s13244-025-02058-7 (PMC12356813; doi:10.1186/s13244-025-02058-7)
Supplement: Supplementary file 1 — ELECTRONIC SUPPLEMENTARY MATERIAL [file 13244_2025_2058_MOESM1_ESM.pdf]

Prognostic and Predictive Imaging Markers of Hepatocellular Carcinoma: A Pictorial Essay

ELECTRONIC SUPPLEMENTARY MATERIAL

Appendix 1

Sample template A

**Observation [#]** – A [size] [mm/cm] observation in segment [Couinaud segment] (series [#], image [#]), with [no] arterial phase hyperenhancement, [no] washout appearance, and [no] capsule appearance. Ancillary features include: [none / list all ancillary features]. **LR-[category].**<br>Poor prognostic features (investigational) include: [large size / washout / peritumoral arterial phase hyperenhancement / HBP hypointensity / tumor necrosis / nonsmooth tumor margin / low ADC / peritumoral HBP hypointensity / bile duct invasion / satellite nodules].<br>Favorable prognostic features (investigational) include: [small size / fat in mass / T1 isointensity / HBP isointensity / HBP hyperintensity / T1 hyperintensity].

Sample template B

**Observation #:**	1 / 2 / 3 / 4 / 5
**Location:**	Segment I / II / III / IVa / IVb / V / VI / VII / VIII
**Size:**	[ ] x [ ] [mm/cm] (image # [ ], series [ ])
**APHE:**	[Yes / No]
**Threshold Growth:**	[Yes / No / N/A]
**PVP/DP Washout Appearance:**	[Yes / No]<br>[HBP phase: Hypointense / Isointense / Hyperintense]
**Capsule Appearance:**	[Absent / Present]
**Ancillary Features:**	[None / *List all that apply]*
**Overall Assessment:**	**LI-RADS category [NC / 1 / 2 / 3 / 4 / 5 / TIV / M]**
**Prognostic features (investigational):**	
**Poor prognosis:**	[Large size / washout / peritumoral arterial phase hyperenhancement / HBP hypointensity / tumor necrosis / nonsmooth tumor margin / low ADC / peritumoral HBP hypointensity / bile duct invasion / satellite nodules].
**Favorable prognosis:**	[Small size / fat in mass / T1 isointensity / HBP isointensity / HBP hyperintensity / T1 hyperintensity].
